# Genetically programmable protein-biomineral core-shell nanovectors for enhancing tumor microenvironment-activated chemotherapy

**DOI:** 10.1016/j.mtbio.2025.102754

**Published:** 2025-12-30

**Authors:** Kaiyue Zhang, Xincheng Sun, Ting Ji, Xinchen Shen, Yao Li, Hang Zhao, Xinyi Yang, Hu Li, Wenwen Huang

**Affiliations:** a Centre for Regeneration and Cell Therapy, The Zhejiang University-University of Edinburgh Institute, Zhejiang University School of Medicine, Zhejiang University, Hangzhou 310058, China; b Deanery of Biomedical Sciences, Edinburgh Medical School, College of Medicine and Veterinary Medicine, The University of Edinburgh, Edinburgh, EH8 9XD UK; c Institute of Smart Biomedical Materials, School of Materials Science and Engineering, Zhejiang Sci-Tech University, Hangzhou 310018, China; d Department of Orthopedics of the Second Affiliated Hospital, Zhejiang University School of Medicine, Zhejiang University, Hangzhou 310058, China; e Dr. Li Dak Sum & Yip Yio Chin Center for Stem Cells and Regenerative Medicine, Zhejiang University School of Medicine, Zhejiang University, Hangzhou 310058, China; f State Key Laboratory of Biobased Transportation Fuel Technology, Zhejiang University, Hangzhou, 310027, China; g Biomedical and Health Translational Research Centre of Zhejiang Province, Zhejiang University, Hangzhou, 310003, China

**Keywords:** Recombinant protein design, Biomineralization, Tumor-responsive, Tumor retention, Nanomedicine

## Abstract

Limited chemotherapy efficacy results in frequent treatment failure events in multiple malignant tumors. Because of limited aqueous solubility, short retention time in the tumor, lack of selectivity toward cancerous cells and non-specific toxicity, there is urgent demand for the discovery of innovative cancer drugs with improved efficacy and selectivity. While nanotechnology offers promising solutions for drug delivery, many nanocarriers still face challenges such as premature drug leakage during circulation, insufficient tumor-specific accumulation, and potential off-target toxicity. To address these limitations, we utilize genetically engineered silk-elastin-like proteins (SELPs) as potent tumor-responsive drug carriers. Tumor cells α_v_β_3_ receptor-specific internalizing RGD peptide (iRGD) was encoded into amphiphilic SELP sequences (S2E3i4Y) to form cancer-selective nanoparticles. To minimize the nonspecific uptake and reduce the leakage of loaded doxorubicin (DOX) during blood circulation, calcium phosphate (CaP) shells were fabricated to be the encapsulation layer of the S2E3i4Y-DOX nanoparticles (S2E3i4Y@CaP-DOX), which prevented premature drug leakage, enhanced the therapeutic safety, and minimized toxicity associated with nonspecific delivery. Meanwhile, the acidic-sensitive CaP shells can be decomposed specifically at the tumor sites, initiating the inner S2E3i4Y-DOX couple to α_v_β_3_-expressing cancer cells for improved tumor-targeting and prolonged tumor retention. *In vivo* assays revealed that S2E3i4Y@CaP-DOX successfully achieved an impressive 4T1 tumor inhibition rate of 75.9 %, much higher than free DOX, without side effects. This core-shell SELP-based platform provides a biocompatible, efficient, and sustainable nanoplatform for tumor-responsive drug delivery, offering a promising strategy for enhanced cancer therapy with spatiotemporal precision.

## Graphical abstract

**Figure:**
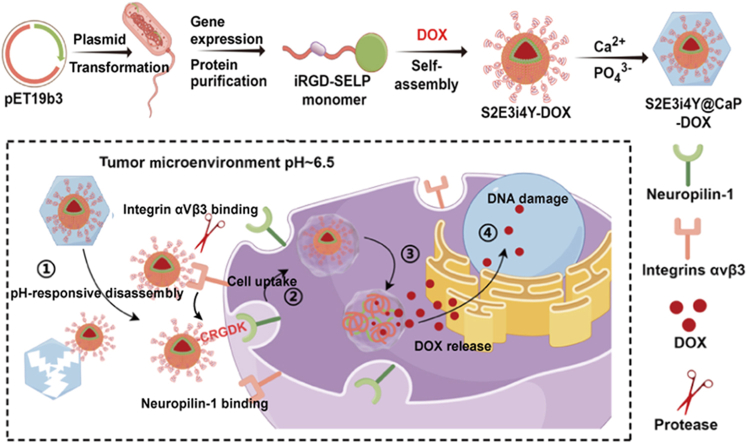

## 1 Introduction

Chemotherapy is widely regarded as a cornerstone intervention in the standard-of-care treatment of neoplastic disease [1,2]. Utilizing cytotoxic agents to destroy tumor cells, chemotherapy has proven effective in providing symptom relief, improving life quality, and extending survival for cancer patients [3]. Besides, chemotherapy serves as a comprehensive combination treatment to enhance the surgical outcomes [4,5], radiotherapy [6,7], and immunotherapy [[8], [9], [10]], and has become the most common tumor therapy regimen [11,12]. However, chemotherapeutic agents still exhibit several limitations in clinical practice [13]. Most chemotherapeutic molecules are hydrophobic, resulting in poor aqueous solubility that complicates intravenous administration [14,15]. Additionally, small-molecule drugs undergo rapid metabolism and clearance, leading to insufficient tumor accumulation. Furthermore, their lack of tumor-targeting specificity causes systemic toxicity, imposing additional burdens on healthy organs [16]. Therefore, advanced delivery technologies need to be invented to improve the therapeutic efficiency and reduce the toxic side effects of chemotherapy drugs.

Functionalization of nanocarriers with tumor cell-affinity ligands to achieve active tumor targeting has been extensively investigated, which strongly enhances the tumor accumulation of loaded agents [17]. The molecules, such as peptides, proteins, and membranes, could be easily modified by various nano-patterns and received satisfactory tumor inhibition efficiency. Pierschbacher and Ruoslahti demonstrated that RGD peptide mediates cell adhesion by binding to integrin receptors (e.g., α_v_β_3_, α_v_β_5_), which are overexpressed in tumor vasculature and metastatic cells [18]. While RGD's tumor-targeting efficiency is constrained by passive diffusion and heterogeneous integrin expression, internalizing RGD peptide (iRGD), a cyclic peptide, exhibits an enhanced tumor-selective accumulation and tumor cell-specific affinity by protease-cleavable design and dual-receptor engagement involving integrin and Neuropilin-1 (NRP-1) [[19], [20], [21]]. This interaction initiates downstream signaling pathways that further facilitate tumor penetration [22,23]. Erkki Ruoslahti et al. modified micelles with iRGD to help nanoparticles penetrate extravascular tumor parenchyma [23,24]. Yoon-Sik Lee et al. designed an iRGD-based monolithic imaging probe for enhanced diagnosis of cancer [25]. Nevertheless, the nonspecific binding of iRGD-modified nanoplatforms to healthy tissues persists, attributable to the constitutive expression of α_v_β_3_ integrin in normal physiological contexts, particularly in the vasculature. This off-target binding exacerbates systemic toxicity (e.g., myelosuppression, hepatotoxicity) and may contribute to multidrug resistance (MDR) via suboptimal drug delivery. In addition, the rapid degradation of iRGD peptides also led to premature leakage of chemotherapeutic agents, which results in tumor nonspecific binding of nanoparticles and challenges the active targeting of nanomedicine to tumors [26]. Therefore, the development of tumor-responsive drug delivery systems is imperative to mitigate nonspecific binding, enhance tumor-targeted accumulation, and minimize off-target toxicity [27].

The tumor microenvironment (TME) exhibits unique biochemical properties, including hypoxia, acidic pH, elevated reactive oxygen species (ROS), aberrant enzyme expression, and high glutathione (GSH) concentrations, which collectively distinguish it from normal tissues [[28], [29], [30]]. These pathological cues offer opportunities for designing TME-responsive drug delivery systems [31]. Among them, pH-sensitive nanocarriers have shown particular promise, exploiting the acidic nature of tumor tissues (pH ∼6.5) and endo/lysosomal compartments (pH ∼5.0–6.0). Common strategies include polymer–drug conjugates with acid-labile linkages (e.g., hydrazone, imine), pH-responsive polymeric micelles such as poly(histidine)-based systems [32], and acid-degradable metal–organic frameworks like ZIF-8 [33]. These platforms remain stable in circulation but undergo structural changes or bond cleavage under acidic conditions, enabling controlled and tumor-specific drug release while minimizing systemic toxicity. The degradation of dissociated molecules *in vivo* remains a critical consideration. In this context, calcium phosphate (CaP) offers a distinct advantage as a biomimetic mineralization strategy, given its intrinsic presence in biological systems [34,35]. Owing to its chemical similarity to native hard tissues (e.g., bones and teeth), CaP exhibits excellent biocompatibility [36]. Furthermore, its metabolic byproducts are naturally compatible with physiological processes, imposing no additional burden on the body [37]. Therefore, TME-responsive surface modification using calcium phosphate may optimize the nanocarrier in terms of particle stability, drug leakage prevention, and biosafety, leading to superior integrated performance [38].

**Scheme 1:**
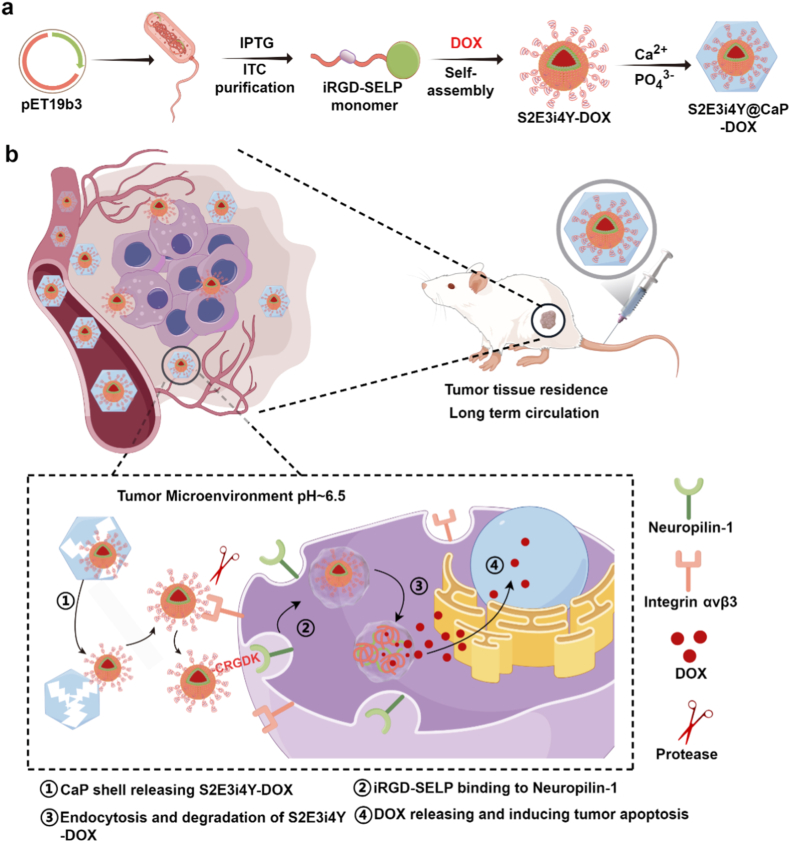
Schematic illustration of S2E3i4Y@CaP-DOX for tumor-responsive cancer treatment. (A) The preparation of S2E3i4Y@CaP-DOX using genetic engineering and chemical modification approaches. (B) The tumor-targeting mechanism of S2E3i4Y@CaP-DOX. S2E3i4Y@CaP-DOX were injected and accumulated at the tumor sites via passive targeting. Then, the CaP shells collapse in the acidic tumor microenvironment, letting the inner S2E3i4Y-DOX core be exposed to the tumor cells and effectively bound to their α_v_β_3_ receptor by iRGD-mediated active targeting. Ultimately, DOX was released and elicited robust tumor chemotherapy.

To lower the nonspecific binding of nanoparticles with iRGD modification, we designed a genetically engineered drug delivery system with precise spatiotemporal drug release behavior through TME-responsive CaP coating (Scheme 1). Silk-elastin-like proteins (SELPs) are amphiphilic biopolymers consisting of alternating hydrophilic elastin-like domains and hydrophobic silk-like domains [[39], [40], [41]]. This molecular architecture facilitates the solubilization and delivery of hydrophobic chemotherapeutics while offering superior biocompatibility and industrial-scale manufacturability [41]. The iRGD gene sequence was genetically inserted into the elastin domains in SELPs, and the iRGD-fused silk-elastin-like proteins (S2E3i4Y) were produced via synthetic biology technology. The chemotherapy agent doxorubicin (DOX) was introduced to the S2E3i4Y proteins and formed S2E3i4Y-DOX nanoparticles via self-assembly. A pH-responsive CaP nanoshell was subsequently deposited onto the S2E3i4Y-DOX core through a biomimetic mineralization process, forming core-shell S2E3i4Y@CaP-DOX nanoparticles. The CaP shell functions as a pH-sensitive ‘lock’ for chemotherapeutics, preventing drug leakage and blocking iRGD-modified nanoparticles from binding to healthy cells, thereby reducing nonspecific uptake during systemic circulation. When the nanoparticles reach the TME, the CaP shell could collapse under the acidic TME and expose the inner S2E3i4Y-DOX. Thus, the hidden iRGD is exposed and coupled to α_v_β_3_-expressing cancer cells, which effectively triggers tumor penetration and tumor cell killing effects. This nanoplatform demonstrates remarkable versatility as a next-generation drug delivery system, exhibiting key characteristics that position it as a highly promising biomedical tool. Its innovative design integrates enhanced drug-loading capacity, controlled release kinetics, and target-specific delivery capabilities, collectively addressing critical challenges in therapeutic delivery. These multifunctional attributes, combined with demonstrated biocompatibility and scalable production potential, highlight its broad applicability across diverse therapeutic contexts.

## 2 Results and discussion

### 2.1 Design and synthesis of S2E3i4Y@CaP-DOX nanoparticles

**Fig. 1:**
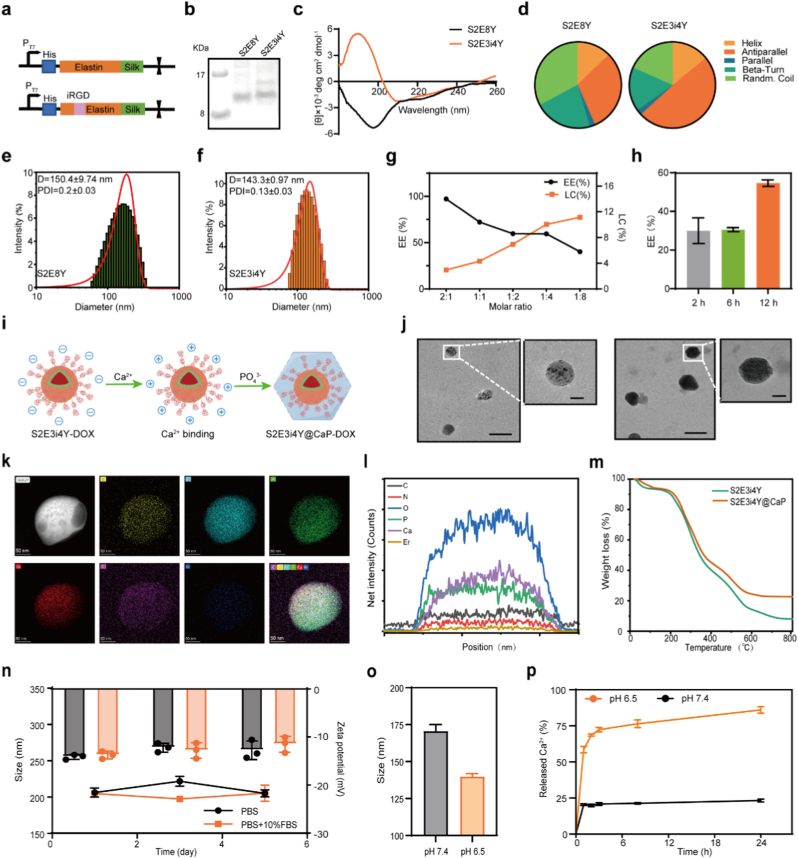
Characterization of S2E3i4Y@CaP-DOX. (a) The protein sequence design of S2E8Y and iRGD-fused S2E3i4Y. (b) SDS-PAGE of purified recombinant proteins. (c) CD spectra and (d) secondary structure content analysis of S2E8Y and S2E3i4Y. Size distribution of self-assembled (e) S2E8Y nanoparticles and (f) S2E3i4Y nanoparticles. (g) The DOX encapsulation efficiency (EE) and loading capacity (LC) under the various molar ratios of S2E3i4Y: DOX after 12 h co-incubation. (h) The encapsulation efficiency of S2E3i4Y-DOX with a molar ratio of 1:2 (S2E3i4Y: DOX) at 2h, 6h, and 12h. (i) Synthesis schematic of core-shell S2E3i4Y@CaP-DOX nanoparticles. (j) Representative TEM images of S2E3i4Y@CaP-DOX before (left) and after (right) calcification. Scale bar = 200 nm or 50 nm. (k) TEM mapping showing the element distribution of S2E3i4Y@CaP-DOX. (l) Element spectra of S2E3i4Y@CaP-DOX. (m) TG curve of S2E8Y and S2E3i4Y@CaP-DOX. (n) The size and zeta-potential of S2E3i4Y@CaP-DOX after incubating with medium at neutral pH. (o) The size and (p) Ca^2+^ release profiles of S2E3i4Y@CaP-DOX at pH 7.4 and pH 6.5 (n = 3). All results are presented as the mean ± SD.

Motivated by the goal of developing an efficient yet biocompatible drug delivery vehicle to realize potent tumor inhibition, recombinant silk-elastin-like protein (SELP) was further functionalized for chemotherapeutic agent delivery. The amphiphilic SELP, which was coded as S2E8Y with sequence (GVGVP)_4_ (GYGVP) (GVGVP)_3_ (GAGAGS)_2_, was previously shown to deliver hydrophobic therapeutics like doxorubicin [41]. Leveraging the intrinsic self-assembly property of SELPs, we further genetically incorporated the iRGD peptide [42], a tumor-penetrating peptide with affinity to α_v_β_3_ integrins, into the protein sequence to form tumor-targeting nanoparticles (Fig. 1a; Fig. S1). The iRGD-fused silk-elastin-like protein was coded as S2E3i4Y with the sequence of (GVGVP)_3_ (CRGDKGPDC) (GVGYP) (GVGVP)_3_ (GAGAGS)_2_. The S2E8Y and S2E3i4Y proteins were expressed in *E. Coli* BL21 Star and purified via the inverse temperature cycling (ITC) method, exploiting the temperature, ionic strength, and pH responsiveness of elastin domains. Successful production and purification of S2E3i4Y was confirmed by SDS-PAGE (Fig. 1b). To investigate the secondary structural conformations of the expressed chimeric S2E3i4Y proteins, circular dichroism (CD) was performed (Fig. 1c–d). The CD spectra of S2E3i4Y showed that the positive ellipticity at 190 nm increased significantly, while the negative signal at 197 nm weakened when compared to S2E8Y. These results suggest that the antiparallel β-sheet content increased while the random coil structure decreased [43], which might be ascribed to the fusion of the iRGD peptide. The α-helical structure of S2E3i4Y appeared to be preserved.

To investigate whether the iRGD-fused SELPs retain the assembly capability of nanoparticles, we measured the size distribution of S2E3i4Y. As shown in Fig. 1e–f, dynamic light scattering (DLS) demonstrated that the average diameters of S2E8Y and S2E3i4Y were 150.37 ± 9.74 nm and 143.33 ± 0.97 nm, respectively. Nanoparticle tracking analysis (NTA) confirmed the uniformity of the nanoparticles in size (Fig. S2). Current research indicates that nanoparticles within the size range of 100–200 nm are optimal for achieving the enhanced permeability and retention (EPR) effect in solid tumors while evading hepatic and splenic filtration [44,45]. Furthermore, nanoparticles with neutral or negatively charged surfaces exhibit prolonged plasma half-lives (> several hours), which is critical for effective tumor accumulation due to prolonged systemic circulation [46]. Therefore, SELP exhibits strong potential as an effective nanocarrier due to its optimal particle size and surface charge characteristics. To examine whether S2E3i4Y can be employed as a potential drug delivery tool, we added the chemotherapeutic drug doxorubicin (DOX) to the S2E3i4Y solution. The drug encapsulation efficiency (EE) and loading efficiency (LC) with different molar ratios of S2E3i4Y: DOX and incubation time were evaluated at room temperature (Fig. 1g–h; Fig. S3). Specifically, the drug encapsulation efficiency (EE) value of S2E3i4Y was measured as 97.27 % and 59.63 % with the proteins to DOX molar ratio at 2:1 and 1:2, while the loading capacity (LC) under the same molar ratios was about 2.95 % and 6.94 % after 12h co-incubation. To optimize the delivery efficiency of DOX, a molar ratio of 1:2 (S2E3i4Y: DOX) was employed in subsequent experimental studies.

To decrease the potential drug leakage and iRGD-mediated nonspecific binding during blood circulation, we further designed a facile strategy to protect the S2E3i4Y-DOX with a temporary “shielding shell” (Fig. 1i). The biomineralized shells of S2E3i4Y@CaP-DOX prevent the binding of nanoparticles to healthy cells and minimize cargo leakage during blood circulation. In contrast, in the acidic tumor microenvironment, the CaP shell is expected to decompose, thereby exposing the inner S2E3i4Y-DOX core to the surrounding cancer cells, binding through iRGD-mediated α_v_β_3_ receptor affinity, and achieving a potent tumor-killing effect. The biomimetic formation of calcium phosphate (CaP) mineralization layers on nanobiomaterials is inspired by natural bone biomineralization, where organic-inorganic interactions guide controlled crystal growth [47,48]. The key principle is that nanostructured organic matrices (e.g., collagen, synthetic polymers, or peptides) provide functional groups (–COOH, –PO_4_^3-^, –OH) that electrostatically bind Ca^2+^ and PO_4_^3−^ ions, initiating nucleation and facilitating the formation of biomineral layers [36,48]. As shown in Fig. 1j–S2E3i4Y@CaP-DOX exhibited a classic core-shell structure with uniform size distribution. TEM mapping confirmed the nanoparticle composition (Fig. 1k). The elements of Ca, P from calcium phosphate, and N, O, C from S2E3i4Y were observed, suggesting the successful coating of the CaP shell. The element intensity further confirmed the co-existence of these components (Fig. 1l). XRD patterns suggested CaP was in its amorphous state (Fig. S4). X-ray photoelectron spectroscopy (XPS) was performed to characterize the elemental composition of the material surface. The Ca 2p, P 2p, and O 1s spectra exhibited well-defined binding energy features that are indicative of calcium phosphate. Notably, the positions and line shapes of these peaks closely match those reported for calcium phosphate coatings in the literature, thereby substantiating the presence of a CaP layer on the surface (Fig. S5) [49]. TG analysis was carried out to evaluate the weight proportion of S2E3i4Y@CaP. As the results shown in Fig. 1m, the CaP shell accounted for about 14.7 % in the prepared nanoparticles. DLS measurement was performed to evaluate the stability of S2E3i4Y@CaP-DOX. We found that the nanoparticles remained stable for at least 5 days under PBS or serum-containing medium (PBS with 10 % FBS) (Fig. 1n; Fig. S6–S7). Meanwhile, the zeta potential also showed no obvious changes. These data indicated the good stability of S2E3i4Y@CaP-DOX under neutral pH, which was suitable for systemic injection.

Subsequently, the pH sensitivity of the prepared S2E3i4Y@CaP-DOX was examined. The DLS results show that the size of S2E3i4Y@CaP-DOX was about 170.7 ± 1.9 nm at pH 7.4 (physiological pH). However, this size decreased to 140.0 ± 4.4 nm after being dispersed in pH 6.5 (acidic pH of TME), which was similar to the naked S2E3i4Y (Fig. 1o). Meanwhile, a weight loss of ∼11.5 % was observed following incubation in acidic conditions (Fig. S8). To further determine the size change of nanoparticles triggered by CaP decomposition, the Ca^2+^ release behavior of S2E3i4Y@CaP-DOX incubating under acidic conditions was monitored. As shown in Fig. 1p, the calcium ion counter suggested that over 87 % of Ca^2+^ was detected at pH 6.5 after 8 h incubation, while only 23 % of the Ca^2+^ was measured in the S2E3i4Y@CaP-DOX solution at pH 7.4. The DOX release profiles from the nanoparticles under different pH conditions (pH 5.0, pH 6.5 and pH 7.4) are compared in Fig. S9. At pH 7.4, the release was relatively slow, with only 29 % of the drug released after 24 h. In contrast, at the acidic pH of 6.5 and 5.0 (mimicking the tumor microenvironment), the nanoparticles exhibited a significantly faster and more extensive release, with over 82 % and 90 % of the payload released within the same time frame. These results together indicated that the manufactured S2E3i4Y@CaP-DOX holds the potential for TME-activated drug delivery, which could stay relatively stable during blood circulation and expose the inner core under the pathologic microenvironment of neoplasms.

### 2.2 Bioactivity of S2E3i4Y@CaP-DOX in vitro

**Fig. 2:**
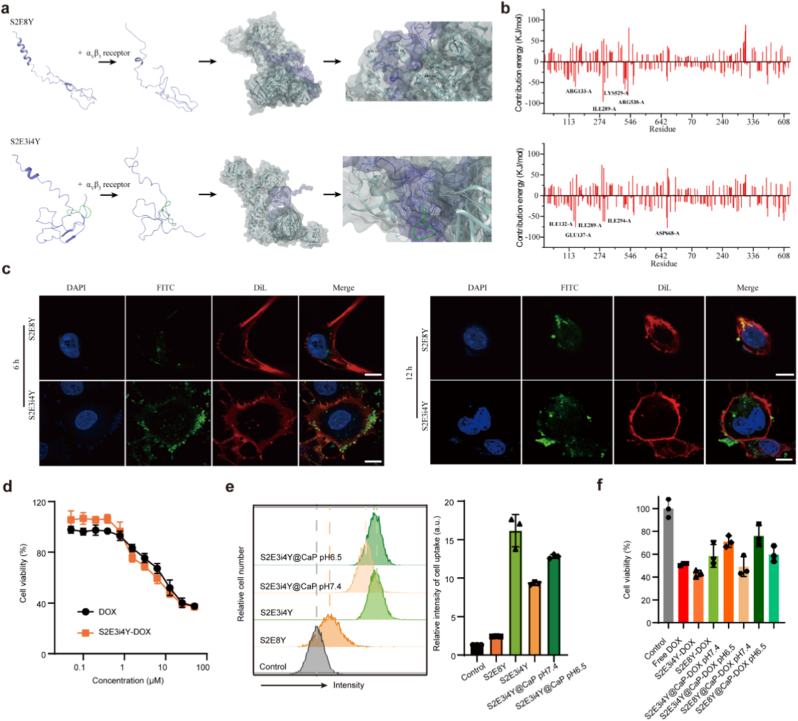
*In vitro* cellular effects of S2E3i4Y@CaP. (a) The overall structure of the iRGD-containing silk elastin-like protein. The iRGD peptide is shown in ribbon form, colored green. (b)Per-residue free energy decomposition diagrams of S2E8Y (top) and S2E3i4Y (down). (c) Cellular uptake of S2E3i4Y@CaP-DOX into 4T1 cells at 6 h and 12 h S2E3i4Y was labeled with FITC (green). The cell membrane and nucleus were stained with DiL (red) and DAPI (blue), respectively. Scale bar = 10 μm. (d) The cell viability of 4T1 after being treated with different concentrations of free DOX and S2E3i4Y-DOX (n = 3). (e) Flow cytometry analysis of the S2E3i4Y@CaP nanoparticles delivery into 4T1 cells under pH 6.5 and pH 7.4 (left). Corresponding quantitative results were also displayed (right). (f) Cytotoxicity of S2E3i4Y@CaP-DOX against 4T1 cells under pH 6.5 and pH 7.4. All results are presented as the mean ± SD. ns, nonsignificant, p > 0.05, ∗p < 0.05, ∗∗p < 0.01. (For interpretation of the references to color in this figure legend, the reader is referred to the Web version of this article.)

Next, we explored the interaction between the α_v_β_3_ receptor and our engineered iRGD ligands to evaluate the bioactivity of S2E3i4Y using molecular dynamics (MD) simulation with GROMACS 2019.6 software. To explain the interaction energy between receptor and ligand, we first used the gmx_mmpbsa script to determine the binding energy of all proteins and ligand complexes in the equilibrium stage. The binding energy of the α_v_β_3_ receptor and S2E3i4Y ligand is shown in Table S1. In the receptor and ligand complex system, the binding free energy of the receptor with fusion S2E3i4Y was lower than that of S2E8Y, which is −577.580 and −465.778 kJ/mol, respectively, indicating their stronger binding interaction. The main interaction energy could be the electrostatic and van der Waals interactions. Fig. 2a displays the overall structures of S2E8Y and S2E3i4Y, respectively, with a green ribbon indicating iRGD in S2E3i4Y. Next, we further decomposed the binding free energy of their interactions. The results revealed that the ILE132-A, GLU137-A, ILE289-A, ILE294-A, and ASP668-A residues in α_v_β_3_ receptor facilitated the affinity interaction between receptor and S2E3i4Y (Fig. 2b).

To investigate the iRGD-mediated cellular selective internalization, the in vitro cell uptake performance of S2E3i4Y was tested. The mouse triple-negative breast cancer (TNBC) cell 4T1 was chosen, and its α_v_β_3_ expression was evaluated using western blotting. Abundant α_v_β_3_ expression was observed in the 4T1 cell membrane compared to cytoplasm, suggesting 4T1 could serve as the potential targeting cell (Fig. S10). We incubated the engineered S2E3i4Y with 4T1 cells for different times and monitored the distribution of nanoparticles using confocal imaging. As shown in Fig. 2c and Fig. S11, FITC-labeled S2E3i4Y exhibited clear green fluorescence around the 4T1 cell surface for 6h incubation, much higher than that in the S2E8Y group. Besides, compared to the S2E8Y, a large proportion of S2E3i4Y located in 4T1 cytoplasm appeared after 12 h incubation, suggesting the efficient iRGD-mediated endocytosis, as evidenced by Dil-labeled 4T1 cell membrane with red fluorescence. Cell uptake of DOX was also studied in Fig. S12. Under neutral conditions, the slow degradation of the calcium phosphate coating resulted in negligible DOX release and consequently undetectable fluorescence over 6 h. In contrast, the acidic environment triggered the rapid disintegration of the coating, exposing the iRGD targeting ligand on the SELP carrier. This led to efficient cellular uptake within 2 h and subsequent nuclear localization of DOX by 6 h.

Next, we evaluated the cytotoxicity of S2E3i4Y using the CCK-8 assay. As shown in Fig. S13, the cell viability of HUVEC remained above 88 % even with a high concentration of S2E8Y and S2E3i4Y at 100 μM, suggesting its good biocompatibility for biomedical applications. Then, the IC50 of DOX-loaded protein nanoparticles (S2E3i4Y-DOX) was determined. The results in Fig. 2d showed that the IC50 value was 13.5 μM for S2E3i4Y-DOX and 16.9 μM for free DOX. The increased cytotoxicity in nano-formulation than the free drug might be ascribed to the specific cellular uptake and prolonged drug release from nanoparticles.

To determine whether the CaP shell would hinder endocytosis, the cell uptake of S2E3i4Y@CaP-DOX was further performed by flow cytometry. As shown in Fig. 2e, limited S2E3i4Y@CaP-DOX endocytosis appeared at pH 7.4, while the S2E3i4Y group exhibited the highest cellular uptake. Notably, at pH 6.5, an increased S2E3i4Y@CaP-DOX internalization by 4T1 cells after 4 h co-incubation occurred. Motivated by this pH-dependent uptake profile, the cell viability of 4T1 after treatment with S2E3i4Y@CaP-DOX under various pH was examined. Consistent with the results of flow cytometry analysis, S2E3i4Y-DOX possessed the greatest cell cytotoxicity. Meanwhile, S2E3i4Y@CaP-DOX at pH 6.5 also exhibited an improved cell-killing effect (Fig. 2f). These data revealed that the prepared S2E3i4Y@CaP-DOX possesses a selective drug release behavior, which could remain stable during systemic circulation and specifically trigger cargo exposure within acidic tumor microenvironments. In addition, Calcium ions released from mineralized nanoparticles in tumor regions are highly likely to cause calcium overload. Calcium overload disrupts intracellular Ca^2+^ homeostasis, triggering mitochondrial damage and endoplasmic reticulum stress that switches cytoprotective autophagy to autophagic/apoptotic cell death. In tumor cells, engineered Ca^2+^-releasing nanomaterials exploit this mechanism by elevating cytosolic Ca^2+^, thereby amplifying the autophagy–apoptosis crosstalk and driving lethal autophagic flux and caspase-mediated apoptosis [[50], [51], [52]].

### 2.3 Tumor targeting and biodistribution of S2E3i4Y@CaP *in vivo*

**Fig. 3:**
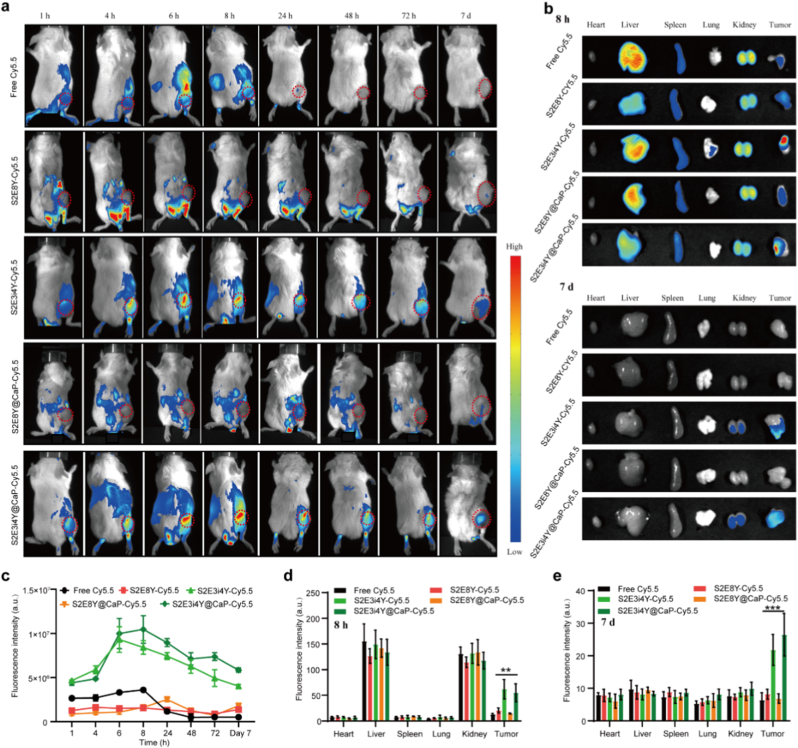
*In vivo* 4T1 tumor targeting of S2E3i4Y@CaP. (a) Time-dependent whole-body distribution of the systemically administered S2E3i4Y@CaP in 4T1 tumor-bearing mice. Images acquired at indicated time points using an *in vivo* imaging system. The red circles indicate the tumor location. (b) The major organs and tumor tissues were separated and imaged to evaluate the accumulation of nanoparticles. (c) The fluorescence intensity changes revealed the targeting effect of S2E3i4Y@CaP nanoparticles. Quantitative analysis of fluorescence intensity of each organ and tumor tissue (d) 8 h and (e) 7 days after S2E3i4Y@CaP injection. All results are presented as the mean ± SD. ∗∗p < 0. 01, ∗∗∗p < 0.001. (For interpretation of the references to color in this figure legend, the reader is referred to the Web version of this article.)

To investigate the *in vivo* tumor targeting ability of S2E3i4Y@CaP vesicles, the nanoparticles were labeled with a Cy5.5 fluorescence probe and systemically injected into 4T1 breast xenografts. As shown in Fig. 3a, the S2E3i4Y-Cy5.5 exhibited reliable fluorescence in tumors after 6 h administration, while limited S2E8Y-Cy5.5 had been observed at the same time, indicating tumor-selective accumulation of S2E3i4Y nanoparticles. The highest fluorescence intensity appeared in the S2E3i4Y@CaP-Cy5.5 group after 8 h of injection (Fig. 3a). Notably, the fluorescence signals were still observed after 7 days in S2E3i4Y-Cy5.5 and S2E3i4Y@CaP-Cy5.5 groups. The biodistribution of S2E3i4Y@CaP in major tissues, including the heart, liver, spleen, lung, kidney, and tumors, was also analyzed. After 8 h of the intravenous administration of the designed nanoparticles, a strong fluorescence appeared in tumor sites both in S2E3i4Y-Cy5.5 and S2E3i4Y@CaP-Cy5.5 groups (Fig. 3b). Besides, the S2E3i4Y@CaP-Cy5.5 remained largely localized in 4T1 tumors even 7 days after treatment, a time when most other groups had been cleared (Fig. 3b).

The average fluorescence intensity analysis further determined a significant enhancement of the accumulation of S2E3i4Y@CaP-Cy5.5, compared to the S2E8Y@CaP-Cy5.5 and S2E8Y-Cy5.5 (Fig. 3c). At 8 h post-injection, the highest fluorescence intensity in the tumor of S2E3i4Y@CaP-Cy5.5 group was around 2.2-fold and 2.4-fold higher than that in the S2E8Y-Cy5.5 and S2E8Y@CaP-Cy5.5 groups, respectively (Fig. 3d). Interestingly, the distribution of S2E3i4Y@CaP-Cy5.5 in 4T1 tumor tissues was a little less than that of S2E3i4Y-Cy5.5, which might be due to the CaP shell delaying its accumulation at the tumor location [53,54]. After 7 days of the formulation administration, the accumulated S2E3i4Y@CaP in the tumors was much higher than that in the S2E3i4Y group (Fig. 3e). As shown in Fig. S14, the group of S2E3i4Y@CaP-Cy5.5 showed a 6.7-fold enhancement in the tumor-to-normal (T/N) ratio at day 7 compared to 8 h, confirming sustained and potent tumor targeting. Taken together, these results indicated that the S2E3i4Y@CaP possesses the tumor-targeting ability, which may be ascribed to both the passive targeting and iRGD-mediated active tumor-specific targeting. Importantly, the S2E3i4Y@CaP remained at the targeted tumors for 7 days, strongly ensuring the efficient therapeutic agent delivery.

### 2.4 S2E3i4Y@CaP-DOX induced anti-tumor effects in TNBC mouse model

**Fig. 4:**
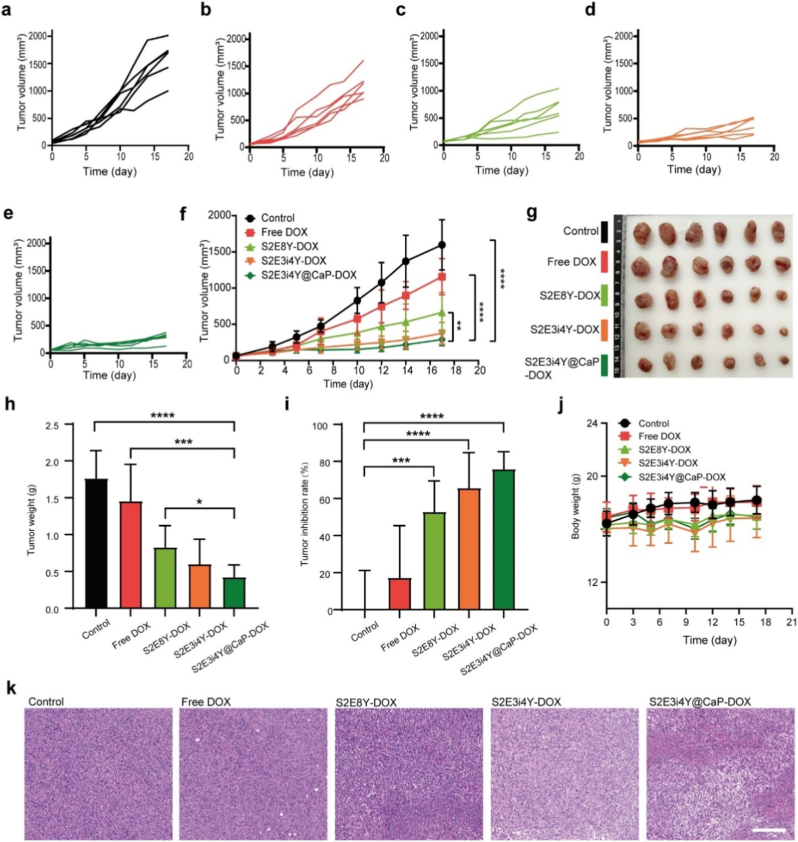
*In vivo* tumor inhibition of S2E3i4Y@CaP-DOX. (a–f) 4T1 tumor growth curve after different treatments (n = 6). (g) Photographs of dissected tumor tissues from each group. (h) 4T1 tumor weight at the endpoint of treatment in various groups (n = 6). (i) The tumor inhibition rate was analyzed based on the results in panel f (n = 6). (j) Mice's body weight during the treatment period. (k) Hematoxylin-eosin (H&E) staining of the treated tumors. Scale bar = 200 μm. All results are presented as the mean ± SD. ∗∗∗p < 0.001, ∗∗∗∗p < 0.0001.

Inspired by the positive results from the tumor targeting results, further evaluation of the *in vivo* anti-tumor response of S2E3i4Y@CaP-DOX was performed. First, the Balb/c mice were subcutaneously injected with 4T1 breast cancer cells to develop the TNBC-bearing animal model. Following the establishment of the tumor model, mice were intravenously administered PBS, free DOX, S2E8Y-DOX, S2E3i4Y-DOX, or S2E3i4Y@CaP-DOX nanoplatforms at equivalent DOX doses. Tumor volume and body weight were monitored throughout the treatment period (Fig. 4a–f). As anticipated, the PBS control group exhibited significant tumor progression, reaching an average volume of ∼1597 mm^3^ by day 17. In contrast, treatment with S2E3i4Y@CaP-DOX markedly suppressed 4T1 tumor growth, yielding an average tumor volume of only ∼290 mm^3^ at the same time point. Meanwhile, mice treated with S2E3i4Y-DOX exhibited an obvious tumor inhibition effect compared to the nontargeted S2E8Y-DOX on the final day (Fig. 4g). The tumor weight results further confirmed the improved anti-tumor response under S2E3i4Y@CaP-DOX treatment (Fig. 4h), attributable to the synergistic combination of the CaP protective function and iRGD-mediated tumor targeting. The tumor inhibition rate in the S2E3i4Y@CaP-DOX treated group was 75.9 % when compared to the control group (Fig. 4i). The mice's body weight showed no significant changes throughout the treatment, suggesting good biocompatibility (Fig. 4j).

Next, the tumors from various treated groups were harvested and analyzed at the end of treatment by H&E staining. As the staining images displayed in Fig. 4k, a sizable number of apoptotic or necrotic cells appeared in the S2E3i4Y-DOX group, and the largest tumor damage was observed in the S2E3i4Y@CaP-DOX treated group. These data were consistent with the tumor growth curves. These results strongly suggest the potent antitumor efficacy of S2E3i4Y@CaP-DOX for TNBC.

### 2.5 Biocompatible assessment of S2E3i4Y@CaP in *Balb/c* mice

**Fig. 5:**
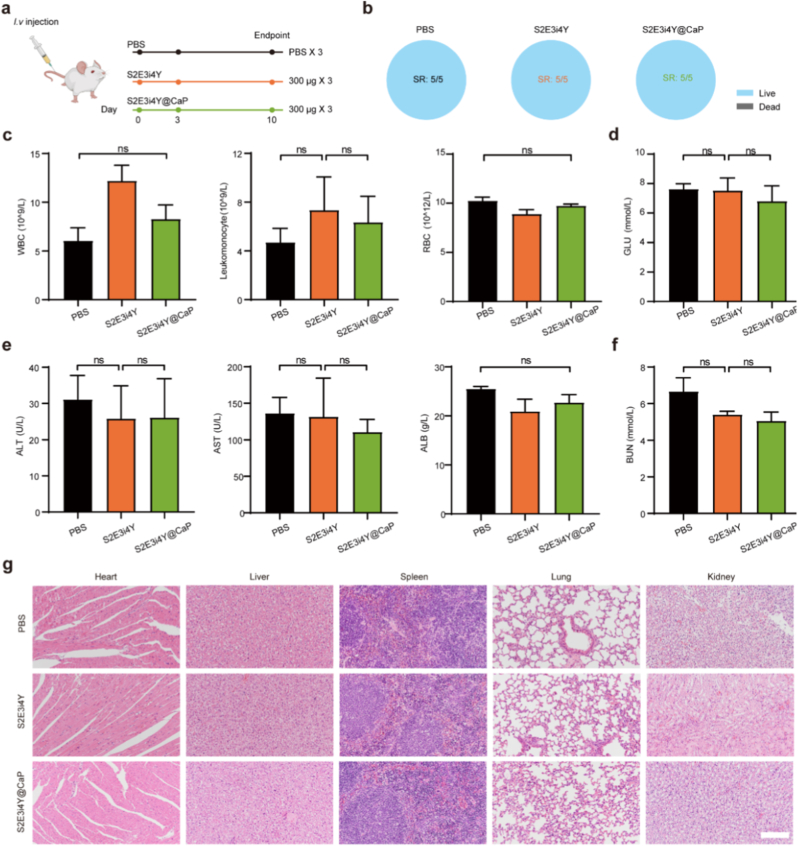
Biosafety evaluation of S2E3i4Y@CaP. (a) Schematic illustration for *in vivo* toxicity evaluation of nanoparticles. Healthy Balb/c mice were systemically injected with S2E3i4Y@CaP on day 0, day 3, and day 10 for three times. (b) Mice's survival rate at the endpoint for different treatment groups. (c) Blood indices in each group after various treatments. WBC - white blood cell, RBC- red blood cell. The serum level of (d) GLU (glucose), (e) ALT (alanine aminotransferase), AST (aspartic transaminase), ALB (albumin), and (f) BUN (blood urea nitrogen) at the indicated groups (n = 3). (g) Hematoxylin-eosin (H&E) staining of the heart, liver, spleen, lung, and kidney collected in mice treated with different formulations. Scale bar = 100 μm. All results are presented as the mean ± SD. ns, nonsignificant, p > 0.05. (For interpretation of the references to color in this figure legend, the reader is referred to the Web version of this article.)

Given that the silk elastin-like protein polymers were produced by engineered bacteria, the *in vivo* biosafety of S2E3i4Y@CaP vesicles should be profiled clearly. As illustrated in Fig. 5a, S2E3i4Y@CaP was administered at days 0, 3, and 10 for three times, as well as 300 μg protein weight for each dose. The vitality of mice was strictly monitored, and survival was recorded. As shown in Fig. 5b, no deaths occurred under high-dose systemic injection during the treatment, suggesting its considerable biosafety. To assess potential hematological toxicity after systemic S2E3i4Y@CaP administration, so as to evaluate whether there is a potential blood-related side effect, a blood routine examination was carried out. As shown in Fig. 5c, the blood indicators, including white blood cell (WBC), leukocyte, and red blood cell (RBC), were analyzed. These data demonstrate minimal hematological impact after the S2E3i4Y@CaP treatment when compared to the control group, suggesting favorable blood biocompatibility of the nanoplatform. Given that the blood biochemistry indicator plays a vital role in evaluating the *in vivo* biocompatibility, the serum in each group was further measured. Fig. 5d–f shows the level of glucose (GLU), alanine aminotransferase (ALT), aspartate aminotransferase (AST), albumin (ALB), and blood urea nitrogen (BUN) in different treatment groups. There were no significant changes across the group, indicating the good biocompatibility of S2E3i4Y@CaP formulations. Furthermore, the major organs' H&E staining shows no obvious histopathological variations after treatment (Fig. 5g; Fig. S15). These results taken together indicate the reliable biosafety of engineered S2E3i4Y@CaP.

## 3 Conclusion

This study successfully developed a novel nano-delivery system, S2E3i4Y@CaP-DOX, based on genetically engineered iRGD peptides and biomimetic mineralization technology, providing an innovative solution to address the critical challenges of poor targeting and severe side effects associated with conventional chemotherapy drugs. The system achieves highly efficient and safe tumor-targeted therapy through a multi-level design. Hydrophobic chemotherapeutic DOX was encapsulated within SELP polymer, significantly improving drug solubility. Additionally, the genetically engineered tumor-penetrating peptide iRGD was incorporated to confer active targeting capability, effectively overcoming the nonspecific targeting limitations of traditional nanocarriers. Furthermore, a biomimetic amorphous calcium phosphate (CaP) shell was constructed, enabling pH-responsive drug release while substantially reducing systemic toxicity via a ‘calcium-phosphate shielding’ effect. Molecular dynamics simulations (GROMACS) and cellular experiments confirmed that the engineered iRGD maintained excellent bioactivity. Both in vitro and *in vivo* studies demonstrated remarkable tumor accumulation and prolonged retention characteristics, with potent antitumor efficacy observed in therapeutic evaluations. Notably, the nanoparticles exhibited reliable biocompatibility even at high-dose administration.

The developed S2E3i4Y@CaP-DOX nanosystem not only provides a safe and efficient solution for chemotherapeutic drug delivery but also establishes a modular design concept that serves as an important reference for developing next-generation nano-delivery platforms. Notably, the core design strategies, such as genetically engineered targeting peptides, and biomimetic mineralization coatings, can be readily adapted to enhance other delivery systems, including liposomes and polymeric micelles. This adaptability positions the platform as a promising universal approach for precision oncology. This multifunctional integrated nano-delivery approach represents the future direction of advanced drug delivery systems, demonstrating significant potential for clinical translation. The combination of genetic engineering with biomimetic material science opens new technological pathways for tumor-specific therapy, offering broad application prospects in personalized medicine and combination therapies.

## 4 Experimental section

### 4.1 Chemicals and reagents

FastBlue Protein Stain solution (Beijing LABLEAD Inc., CHN); Rabbit Anti-ITGB3 Rabbit pAb Polyclonal Antibody (Beijing Biosynthesis Biotech Co., Ltd, CHN); Goat Anti Rabbit IgG H&L (Abcam, CHN); DOX (MCE, CHN); Calcium chloride (Sigma, USA); Potassium hydrogen phosphate (Sigma, USA); Dialysis tube MD44 (Dacheng, CHN); Gel-Red (Beyotime Co., Ltd, CHN); PMSF (Bestbio Biotech Co., Ltd, CHN); Cyanine5.5 NHS ester (DuoFluor, CHN); FITC-NHS (Stargraydye, CHN); DL5000 DNA Marker (Vazyme Biotech Co., Ltd, CHN); LB Borth Power (Sangon Biotech Co., Ltd, CHN); DAPI (Biosharp Biotech Co., Ltd, CHN); Dil (YEASEN Biotech Co., Ltd, CHN).

### 4.2 Synthesis of iRGD-SELP

#### 4.2.1 Construction of expression plasmids

The gene encoding S2E3i4Y with the iRGD fusion region and S2E8Y without any targeting peptide was PCR-amplified from a previously synthesized SELP-containing pUC vector (Sangon Biotech, CHN) and cloned into pET-19b3 (the vector encoding ELP was constructed according to the literature) using Ban II restriction sites. The sequence of S2E3i4Y is composed of an elastin domain (GVGVP)_3_ (CRGDKGPDC)(GYGVP) (GVGVP)_3_ and a silk domain (GAGAGS)_2_; the sequence of S2E8Y is composed of an elastin domain (GVGVP)_4_ (GYGVP) (GVGVP)_3_ and a silk domain (GAGAGS)_2_. The primers utilized have the following design:

Forward primer:5′CAGGGTTTTCCCAGTCACG3’

Reverse primer:5′ GAGCGGATAACAATTTCACAC3’

#### 4.2.2 Protein expression and purification

After verification by DNA sequencing, the constructed plasmid encoding SELP was transformed into *E. coli* strain BL 21 star (DE3) chemically competent cell (Weidi Biotechnology Co., Ltd., Shanghai) and incubated in Luria Bertani (LB) broth medium containing 50 μg/mL ampicillin at 37 °C. The culture was inoculated into 1 L of LB broth medium and shaken at 250 rpm until the optical density at 600 nm (OD600) reached 0.8. At this point, isopropyl-β-D-thiogalactopyranoside (IPTG) was added to a final concentration of 1 mM to induce expression.

The S2E3i4Y peptide was purified using an inverse transition cycling (ITC) approach. Briefly, *E. coli* cells were centrifuged at 8500 rpm for 15 min at 4 °C to collect the pellets, which were then resuspended in PBS and disrupted using a homogenizer (AH-NANO, ATS Engineering Inc., CHN). The resulting cell lysate was centrifuged at 10000 rpm for 30 min at 4 °C. To the resulting supernatant, 9 g of sodium chloride was added, followed by incubation at 90 °C for 2 h. After incubation, the mixture was centrifuged at 10000 rpm for 5 min at 40 °C to collect the pellet containing SELP, which was subsequently recovered by incubation in deionized water for 12 h at 4 °C. The supernatant was then dialyzed against deionized water using a membrane with a molecular weight cutoff (MWCO) of 2 kDa for 24 h. The purity of the protein was assessed using SDS-PAGE.

### 4.3 Preparation of nanoparticles

Firstly, the purified protein was dissolved in PBS, mixed, and prepared as a 1.6 mg/mL solution. Then, DOX ethanol solution was added to SELP aqueous solution and dialyzed with a 2000 Mw dialysis membrane. After dialysis, its final concentration was calibrated to 0.1 mg/mL by UV absorbance. The final samples were called SELP-DOX. The encapsulation efficiency of doxorubicin was calculated using the following equation:

$$\%EE\;of\;DOX=100\times \frac{incorporated\;DOX}{initial\;DOX\;added}$$

The loading capacity of doxorubicin was calculated using the following equation:

$$\%LC\;of\;DOX=100\times \frac{Total\;amount\;of\;DOX-Free\;amount\;of\;DOX}{nanoparticles\;weight}$$

Secondly, this nanocarrier was mineralized with calcium phosphate. An equal volume of DMEM was added to the protein solution and equilibrated at 4 °C overnight. Add 1 volume of calcium chloride solution (CaCl_2_, 18 mM, pH 8), mix well, and equilibrate at room temperature for 1 h. Then add 2 vol of potassium hydrogen phosphate solution (K_2_HPO_4_, 4.2 mM, pH 7.4) and incubate for 6 h at 37 °C. Finally, the sample was centrifuged at 10,000 rpm for 10 min and the precipitate was taken. The precipitate was resuspended with PBS and the sample was called SELP@CaP-DOX.

### 4.4 Dynamic light scattering (DLS)

DLS was carried out on a Litesizer 500 instrument (Anton Paar, Trading Co.Ltd, Shanghai.) equipped with a temperature controller. All samples were filtered through a 0.45 μm Millipore filter before measurement. The protein solutions (0.1 mg/mL in water) were introduced into the Omega cuvette, and the samples were stabilized at 25 °C for 5 min prior to measurement. To obtain the average size and size distribution, the intensity autocorrelation functions were analyzed using the Dynamics software (Anton Paar, Trading Co.Ltd, Shanghai).

### 4.5 Circular dichroism (CD)

CD spectra were recorded, ranging from 200 nm to 260 nm, on a Pistar π-180 (Applied Photophysics Ltd) instrument. Samples were diluted to a concentration of 0.15 mg/mL in H_2_O. The CD spectra, analyzed with the DICHROWEB program packages, yield the percentages of *α*-helices, *β*-sheets, turns and random coil in the secondary structure of SELP.

### 4.6 X-ray photo-electron spectroscopy (XPS)

XPS was conducted to analyze the surface elemental composition and chemical states of the samples. The measurements were performed using a Thermo Scientific K-Alpha (Thermo Scientific, America) equipped with a monochromatic Al Kα X-ray source (hν = 1486.6 eV). The samples were synthesized and vacuum-dried at RT before the test. Survey spectra were recorded to identify the overall elemental composition, followed by high-resolution scans of Ca 2p, P 2p, and O 1s to determine detailed chemical states. Charge neutralization was applied during acquisition when necessary. All binding energies were calibrated using the C 1s peak at 284.8 eV as an internal reference. Data processing and peak fitting were performed using XPSPEAK41.

### 4.7 Molecular dynamics simulations

Molecular dynamics (MD) simulations of the protein-ligand complex were performed to explore the interaction between the receptors and ligands by using GROMACS 2019.6 software [55]. The amber99sb-ildn force field was used to generate the parameters and topology of proteins. Setting the size of the simulation box so that the distance between each atom of the protein and the box was greater than 1.0 nm. Filling the box with an explicit solvent-simple point charge model (SPC216 water molecules) and replacing the water molecules with Na^+^ and Cl^−^ counterions to make the simulation system electrically neutral. The entire system was optimized by the steepest descent method, so that the unreasonable contact or atom overlap in the system is reduced. To achieve sufficient pre-equilibration of the simulation system, the NVT ensemble and the NPT ensemble were performed for 100 ps at 300 K and 1 bar, respectively. Subsequently, the MD simulation of 50 ns was performed with periodic boundary conditions, and the temperature (300 K) and pressure (1 bar) were controlled by the V-rescale and Parrinello-Rahman methods, respectively [56]. The Newton equation of motion was calculated using the leapfrog integration with a time step of 2 fs. The long-range electrostatic interaction was calculated by the Particle Mesh-Ewald (PME) method using Fourier spacing of 0.16 nm, and the LINCS method was used to constrain all bond lengths. The visual molecular dynamics (VMD) software 1.9.3 version and PyMOL 2.4.1 version were used to display, analyze, and animate trajectories visually [57]. The binding free energy of the compound was calculated by gmx_mmpbsa (http://jerkwin.github.io/gmxtools/).

### 4.8 Cell culture

4T1 and HUVEC cells were purchased from Pricella Life Science & Technology Co., Ltd (CHN). 4T1 cells were cultured in RPMI-1640 medium containing 50 U per mL streptomycin, 100 U per mL penicillin, and 10 % fetal bovine serum (FBS, Gibco, USA). HUVEC cells were cultured with DMEM medium containing 50 U per mL streptomycin, 100 U per mL penicillin, and 10 % FBS. The cells were cultured at 37 °C in a humidified incubator with 5 % CO_2_. All the cell cultures were maintained in 10 cm culture dishes for use.

### 4.9 Cell viability

The cell viability of samples to 4T1and HUVEC in vitro was evaluated by CCK8 assay, which was based on the number of living cells reacting with CCK8 to form formazan. The cells were seeded at a density of 5000 cells/well into 96-well plates. After 24 h incubation, the culture medium was replaced with the medium containing a series of solutions of DOX, S2E3i4Y, S2E3i4Y-DOX, and S2E8Y-DOX nanoparticles with concentration gradients, and then incubated at 37 °C for 24 h. 10 μL CCK8 assay was added in each well and incubated at 37 °C for 1 h. The absorbance value was determined at 480 nm in a microplate reader (Tecan, SPARK multimode reader, Austria). The percentage of viable cells following DOX, S2E3i4Y, S2E3i4Y-DOX, and S2E8Y-DOX nanoparticle treatments was determined relative to a control group (PBS). The data was obtained from three parallel wells per assay, and the assay was performed at least three times.

The cell viability of nanoparticles under acid environment was measured. The materials was pretreated with pH7.4 or pH6.5 PBS buffer for 12 h. This pretreated formulation was then diluted into the complete culture medium. The cells were seeded at a density of 5000 cells/well into 96-well plates. After 24 h incubation, the culture medium was replaced with the medium containing pretreated formulation, and then incubated at 37 °C for 24 h. 10 μL CCK8 assay was added in each well and incubated at 37 °C for 1 h. The absorbance value was determined at 480 nm in the same microplate reader.

### 4.10 Animal experiments

All animal studies were carried out in compliance with the guidance principles of the care and use of laboratory animals and an approved protocol from the Animal Ethics Committee of Zhejiang University (ZJU20220518). The animals were housed in a specific pathogen-free facility with free access to food and water at the Research Animal Resources facility of Zhejiang University. Female BALB/c mice (5 weeks old; weight around 20 g) were purchased from Vital River Laboratory Animal Technology Co., Ltd. (Beijing, CHN). The 4T1 orthotopic tumor model was generated by the injection of 5 million 4T1 cells into the breast of per female mouse. The tumor would be ready after one week of injection. The tumor diameters were measured with calipers every two days, and tumor volumes were calculated as V_tumor_ = width^2^ × length/2. After one-week post-tumor inoculation, tumor size reached around 5 mm^3^ in volume.

### 4.11 In vivo targeting & anti-tumor assay

The homing procedure was performed in 4T1 tumor-bearing mice. The SELPs were marked by NHS-cy5.5 fluorescence molecular via click reaction. Mice were randomly divided into 5 groups (free Cy5.5, S2E8Y-Cy5.5, S2E3i4Y-Cy5.5, S2E8Y@CaP-Cy5.5, and S2E3i4Y@CaP-Cy5.5). Various drug was injected into the mice via a one-time tail vein intravenous injection at a 10 μg/kg dosage of Cy5.5. After 1 h/4 h/6 h/8 h/24 h/48 h/72 h/7 days, mice were anesthetized and then fluorescently imaged with a small animal *in vivo* imager (Biospace, PhotonIMAGER™, USA). And mice were euthanized and the tissues were harvested after the injection at 8 h/7 days. The fluorescence intensity of tissues was measured by the same machine.

For the long-term efficacy, the tumor growth of the 4T1 model was monitored for 17 days after three times of treatments in the first week via tail vein intravenous injection at a 3 mg/kg dosage of DOX. The mice were randomly divided into groups (n = 5 per group) receiving different injections as follows: 1) PBS, 2) free DOX in PBS, 3) S2E8Y-DOX nanoparticles, 4) S2E3i4Y-DOX nanoparticles, 5) S2E3i4Y@CaP-DOX biomineralized nanoparticles. Tumor volume and body weight were recorded. The obtained tumors were accurately weighed. The harvested tumors were weighed, fixed, processed, and embedded in paraffin for use.

### 4.12 Serum biochemical and hematological characteristics

To test the biosafety of the de novo designed nano delivery material, blood was collected after 7 days of the second treatment with SELP. Blood samples were taken from the mice's eyes and collected into a 2 mL anticoagulation tube. The whole blood was stored in the ice and did the hematological characteristics in 2 h. Serum was collected after the 1500 rpm centrifugation for 10 min. Serum aspartate aminotransferase (AST) and alanine aminotransferase (ALT), albumin (ALB) blood urea nitrogen (BUN) levels were measured using assay kits.

### 4.13 Statistical analysis

All quantitative results were presented as mean ± standard deviation (SD). Non-paired t-tests were used for the statistical analysis of comparison between two groups, and one-way analysis of variance (ANOVA) was used for comparison among multiple groups. Data analysis was performed using GraphPad Prism software (GraphPad Software, USA). Significance was defined as ∗∗∗p < 0.001 and ∗∗p < 0.01.

## CRediT authorship contribution statement

**Kaiyue Zhang:** Writing – review & editing, Writing – original draft, Validation, Methodology, Investigation, Formal analysis, Data curation, Conceptualization. **Xincheng Sun:** Validation, Investigation. **Ting Ji:** Methodology, Investigation. **Xinchen Shen:** Methodology, Investigation. **Yao Li:** Writing – review & editing, Methodology. **Hang Zhao:** Investigation. **Xinyi Yang:** Investigation. **Hu Li:** Investigation. **Wenwen Huang:** Writing – review & editing, Supervision, Resources, Project administration, Funding acquisition, Conceptualization.

## Declaration of competing interest

The authors declare the following financial interests/personal relationships which may be considered as potential competing interests: Wenwen Huang reports financial support was provided by Huadong Medicine Joint Funds of the Zhejiang Provincial Natural Science Foundation of China. Wenwen Huang reports financial support was provided by National Natural Science Foundation of China. If there are other authors, they declare that they have no known competing financial interests or personal relationships that could have appeared to influence the work reported in this paper.

## Data Availability

Data will be made available on request.
