# Supplementary material for: Genetically programmable protein-biomineral core-shell nanovectors for enhancing tumor microenvironment-activated chemotherapy
Source: Mater Today Bio. 2025 Dec 30;36:102754. doi: 10.1016/j.mtbio.2025.102754 (PMC12813328; doi:10.1016/j.mtbio.2025.102754)
Supplement: Multimedia component 1 [file mmc1.pdf]

Supporting Information

Genetically Programmable Protein-Biomineral Core-Shell Nanovectors for Enhancing Tumor Microenvironment-Activated Chemotherapy

*Kaiyue Zhang[1], Xincheng Sun[1], Ting Ji[1], Xinchen Shen[1,2], Yao Li[3], Hang Zhao[1], Xinyi Yang[1], Hu Li[1] and Wenwen Huang[1,2,4,5,6,7]**

[1] Centre for Regeneration and Cell Therapy, The Zhejiang University-University of Edinburgh Institute, Zhejiang University School of Medicine, Zhejiang University, Hangzhou 310058, China

[2] Deanery of Biomedical Sciences, Edinburgh Medical School, College of Medicine and Veterinary Medicine, The University of Edinburgh, Edinburgh, EH8 9XD UK

[3] Institute of Smart Biomedical Materials, School of Materials Science and Engineering, Zhejiang Sci-Tech University, Hangzhou 310018, China

[4] Department of Orthopedics of the Second Affiliated Hospital, Zhejiang University School of Medicine, Zhejiang University, Hangzhou 310058, China

[5] Dr. Li Dak Sum & Yip Yio Chin Center for Stem Cells and Regenerative Medicine, Zhejiang University School of Medicine, Zhejiang University, Hangzhou 310058, China

[6] State Key Laboratory of Biobased Transportation Fuel Technology, Zhejiang University, Hangzhou, 310027, China

[7] Biomedical and Health Translational Research Centre of Zhejiang Province, Zhejiang University, Hangzhou, 310003, China

*Corresponding authors

E-mail: wenwenhuang@intl.zju.edu.cn

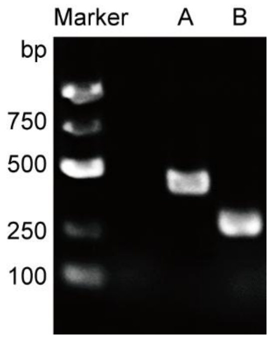

Figure S1. The gel electrophoresis results of plasmid PCR (A) with S2E3i4Y monomer and (B) without S2E3i4Y monomer.

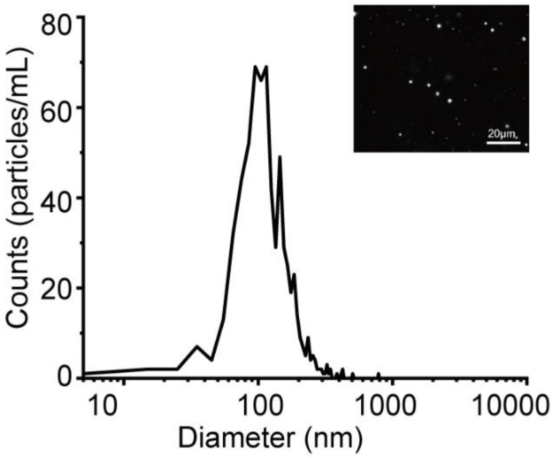

Figure S2. Size and image of S2E3i4Y nanoparticles using NTA.

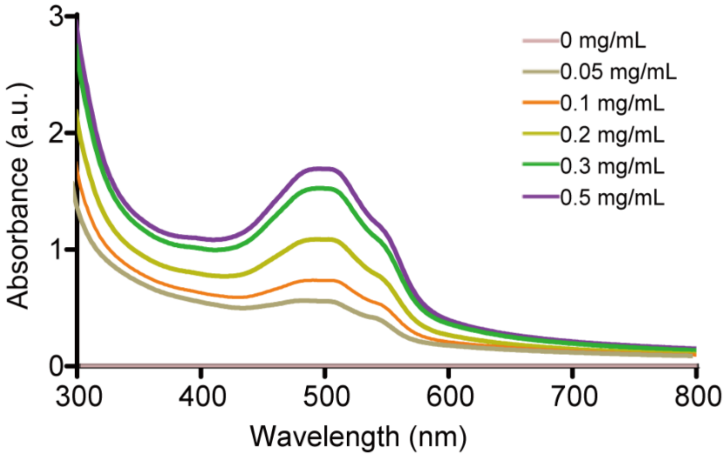

Figure S3. The UV-vis spectrum of S2E3i4Y-DOX with various concentrations of DOX.

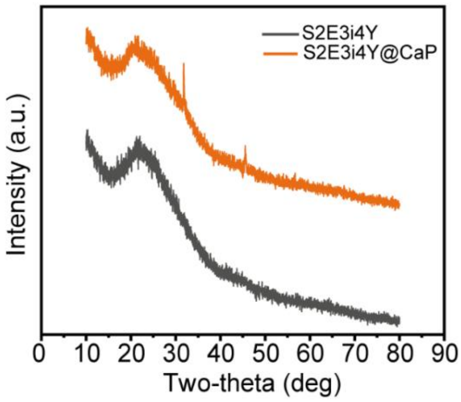

Figure S4. XRD spectrum of S2E3i4Y and S2E3i4Y@CaP.

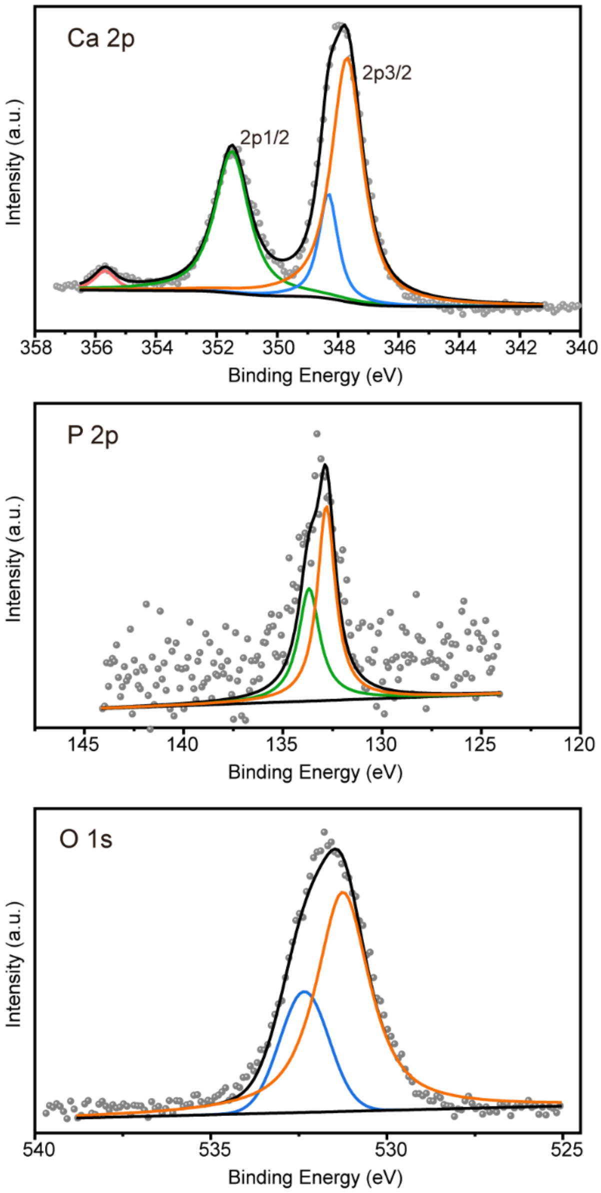

Figure S5. XPS spectrum of S2E3i4Y@CaP.

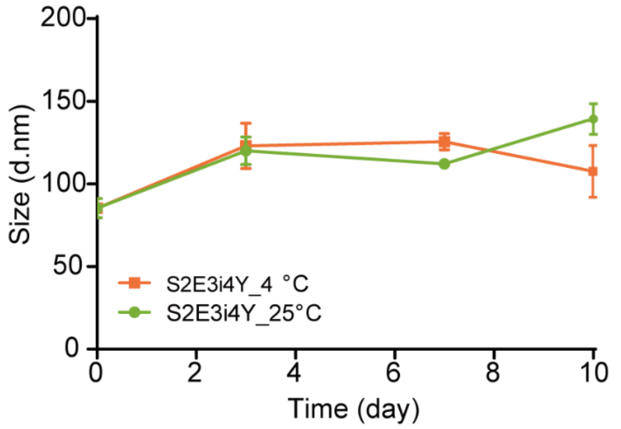

Figure S6. The size and stability of S2E3i4Y at 4°C and 25°C over 10 days.

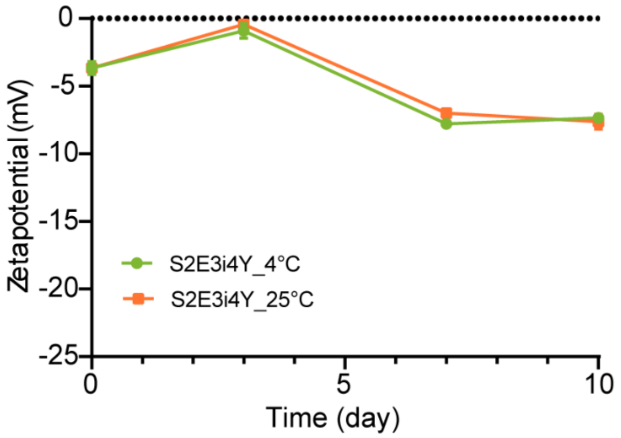

Figure S7. The surface charge and stability of S2E3i4Y at 4°C and 25°C over 10 days.

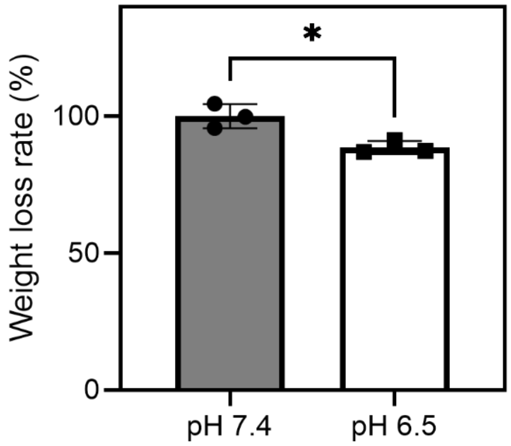

Figure S8. The weight loss of S2E3i4Y@CaP-DOX at pH 7.4 and pH 6.5 over 24 h.

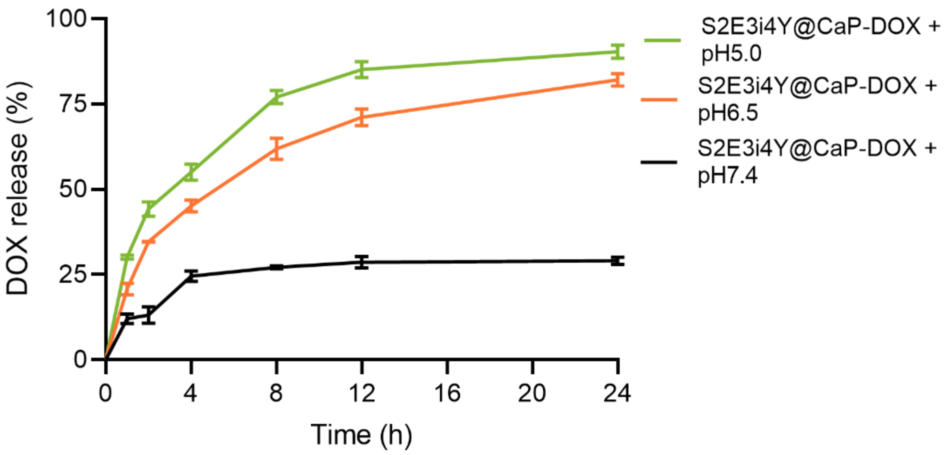

Figure S9. The DOX release kinetics under different pH (7.4, 6.5, 5.0).

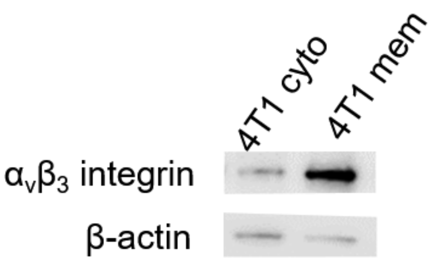

Figure S10. The Western Blot images of $\alpha_v\beta_3$ expression in the 4T1 cell cytoplasm and 4T1 cell membrane.

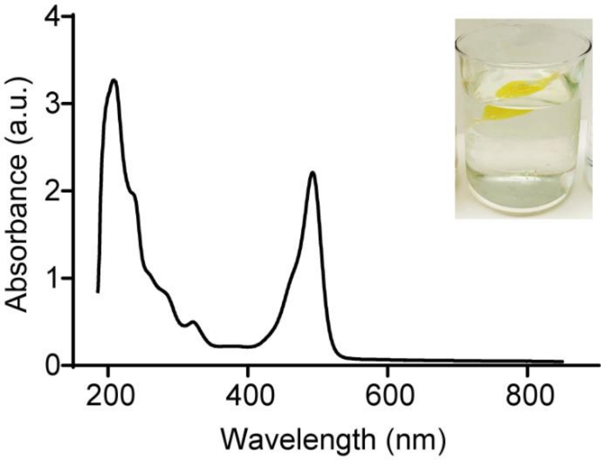

Figure S11. The UV-vis spectrum and dialysis image of S2E3i4Y labeled with FITC.

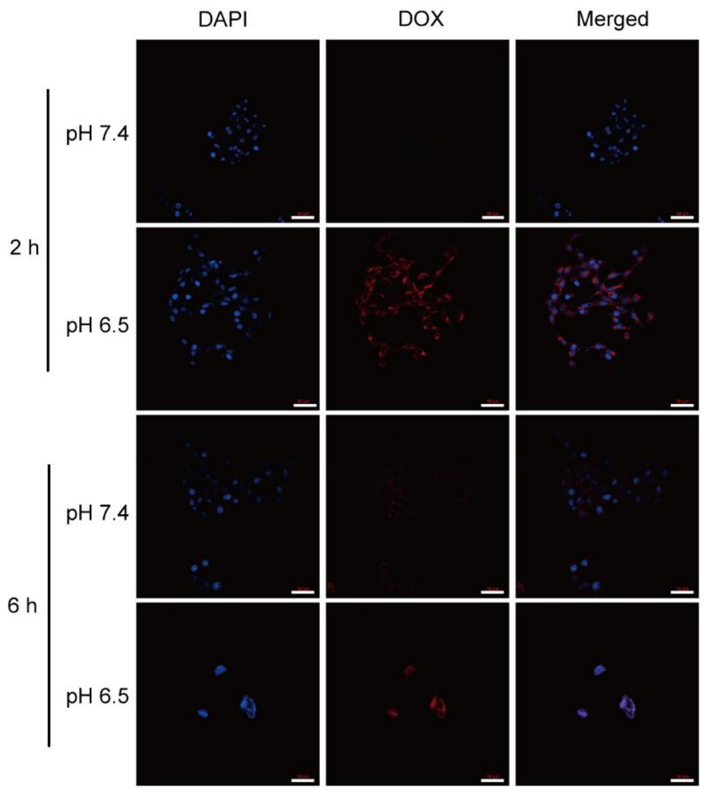

Figure S12. Delivery of doxorubicin into 4T1 cells via S2E3i4Y@CaP-DOX treated at pH 7.4 or pH 6.5 by CLSM. The nucleus was stained with DAPI (blue signal). The scale bar is 50 μm.

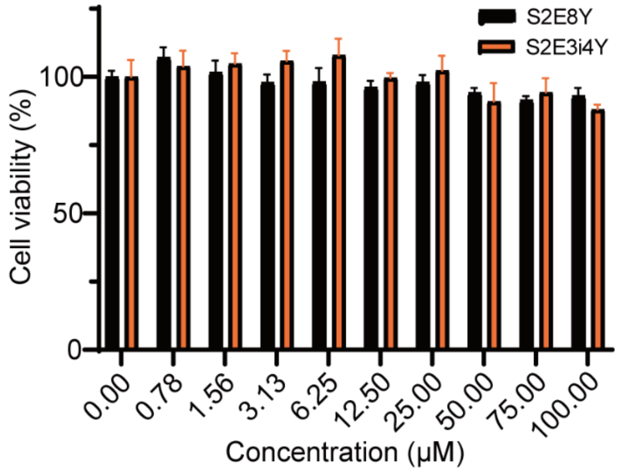

Figure S13. The CCK-8 assay of HUVEC.

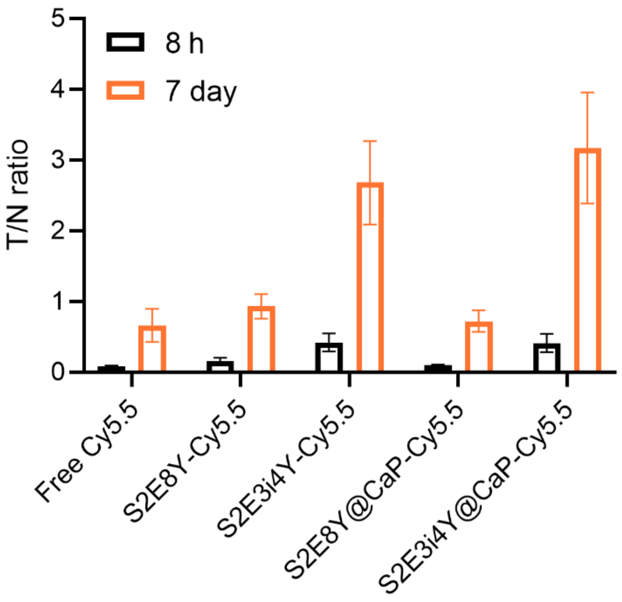

Figure S14. The tumor-to-normal (T/N) ratio based on the fluorescence intensity in small animal imaging.

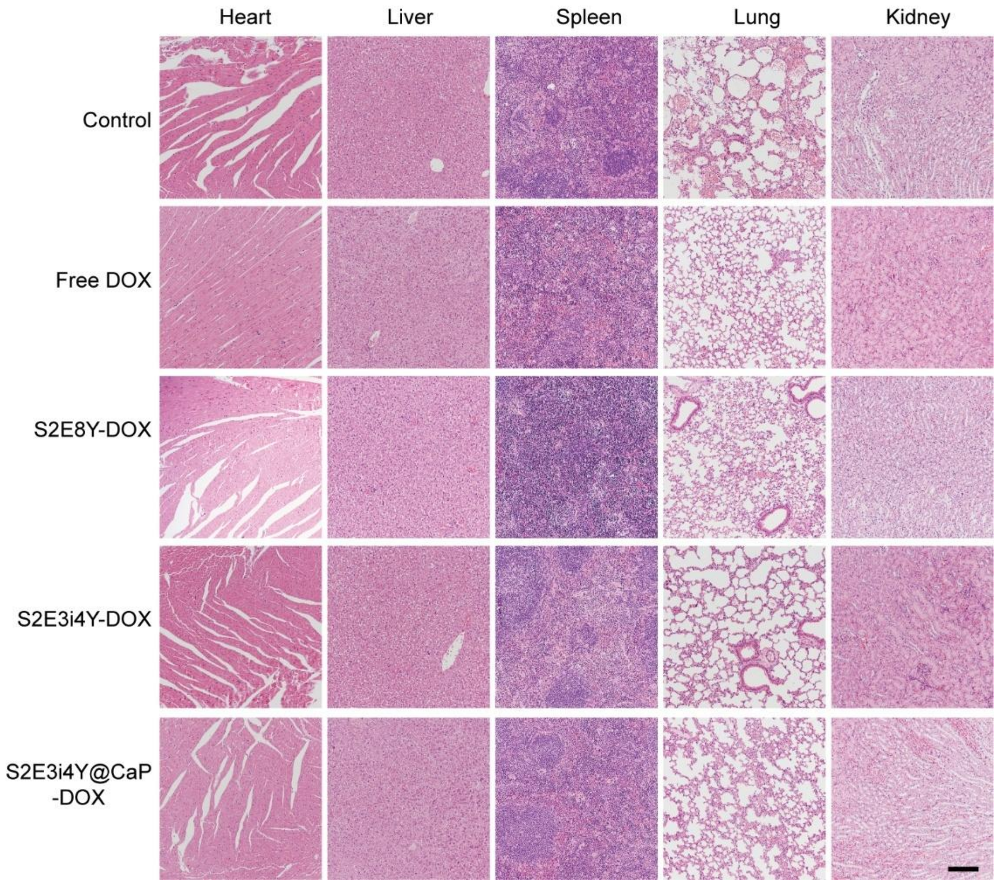

Figure S15. H&E staining of the heart, liver, spleen, lung, and kidney collected in mice treated with different formulations. Scale bar = 100 μm.

Table S1. MMPBSA analysis of $\alpha_V\beta_3$ receptor and S2E3i4Y ligand.

Energy	S2E3i4Y	S2E8Y
Van der Waals energy (KJ/mol)	-673.693	-741.477
Electrostatic energy (KJ/mol)	-1002.712	-627.234
Polar solvation energy (KJ/mol)	1073.644	872.955
Nonpolar solvation energy (KJ/mol)	-88.287	-86.249
Total binding energy (KJ/mol)	-691.048	-582.005
-TΔS (KJ/mol)	113.467	116.227
Total binding free energy (KJ/mol)	-577.580	-465.778
